# Preparation of *β*-lactoglobulin-derived tryptophan peptide and its effect on anxiety-like behaviors in *Zebrafish*

**DOI:** 10.3389/fnut.2022.1100718

**Published:** 2023-01-06

**Authors:** Xiping Zhu, Dan Xie, Qiong Zhu, Yufeng Li, Chun Cui

**Affiliations:** ^1^College of Biological and Food Engineering, Anhui Polytechnic University, Wuhu, China; ^2^School of Food Science and Engineering, South China University of Technology, Guangzhou, China; ^3^Research Institute for Food Nutrition and Human Health, Guangzhou, China

**Keywords:** β-lactoglobulin, tryptophan peptides, zebrafish, behavior, serotonin

## Abstract

This study aimed to obtain three Trp-containing peptides from *β*-lactoglobulin and study their effects on anxiety-like behaviors in zebrafish. Three Trp-containing peptides were prepared from *β*-lactoglobulin by selective enzymatic hydrolysis and identified by UPLC-Q-TOF MS/MS. The anxiety-like behaviors of zebrafish were reduced after two weeks of administrated of *β*-lactoglobulin Trp peptides (LAWP), VAGTWY, VAGTW and G TW(concentration of 56 μg/mL or 500 μg/mL). As an index of serotonergic activity, we assessed the enhancing abilities of 5-HT synthesis. The treatment remarkably enhanced the 5-HT synthesis by upregulation of Trp concentration and Trp hydroxylase activation. In addition, this study further validated the anti-anxiety effects of whey protein hydrolysate with a high Trp index in animal and the experimental results were consistent with those reported in previous studies. Our results showed that *β*-lactoglobulin Trp peptides ingestion has a significant anti-anxiety effect as evidenced by the increasing Trp concentration, TPH activation and 5-HT level compared to the control group, with the VAGTW being the more effective.

## 1. Introduction

Anxiety disorder is a psychiatric disorder caused by feelings of excessive, exaggerated, and extended anxiety. Its incidence is increasing with the trend of urbanization and lifestyle changes, which affect human health and life negatively and widely (1). So, it is a problem that needs to be solved urgently in public health due to the complex pathogenesis and difficult clinical medication of anxiety (2, 3). According to these previous studies, most anti-anxiety drugs (serotonin-norepinephrine reuptake inhibitors and selective serotonin reuptake inhibitors) act by influencing certain brain circuits and the neurotransmitter dynamics (such as the increase in the levels of 5-HT in the synaptic cleft) in the brain (4, 5). 5-HT is a monoaminergic neurotransmitter with high value that plays an important role in the treatment and regulation of anxiety in both humans and animals (6). However, the rate of 5-HT biosynthesis is dependent on tryptophan hydroxylase (TPH) activation and Trp availability (7). TPH is the rate-limiting enzyme in 5-HT synthesis, which catalyzes the conversion of Trp to 5-hydroxytryptophan (5-HTP) and then to 5-HT (8, 9). The availability of Trp is affected by the five large neutral amino acids (5LNAAs, namely, Val, Ile, Leu, Phe, and Tyr, which compete with Trp during its passage through the blood–brain barrier by carriers) (10). However, these clinical medicines have their own limitations and present strong side effects (obesity, drowsiness, sexual dysfunction, and so on) and safety concerns (the mechanism of action is not clear) (5).

In recent years, nutrition intervention has been used globally as an alternative medicine for the treatment of anxiety. Bioactive peptides may deserve attention because peptides have exhibited high nutritional values, effectiveness, low side effects, and high absorption efficiency (dipeptides/tripeptides > free amino acids) (10, 11). Numerous studies showed that the nutritional factor Trp and its peptides have been used to reduce anxiety-like states and unavoidable stress (like those resulting from frustration) (4, 10, 12). Studies showed that Trp has a positive effect on anti-anxiety behavior and reduces post-stress plasma cortisol concentrations in vertebrates (including teleosts) (7). The sensitivity of mood was improved, and the cortisol was reduced by Trp supplementation, during stressful challenges in women (13). A high ratio of Trp/5LNAAs ingestion increases Trp access to the brain, leading to enhanced brain-derived 5-HT synthesis and providing anti-anxiety-like effects in animals (10, 14). The results of Zhu et al. (4, 10) show that dietary supplementation with Trp oligopeptide (γ-[Glu]n-Trp) reversed anxiety behavioral dysfunctions and increased the activity of TPH in the hypothalamus, the hippocampus, and the prefrontal cortex. In addition, whey protein hydrolysate with a high ratio of Trp/5LNAAs reversed anxiety behavioral dysfunctions in mice (10). *β*-lactoglobulin is the most abundant protein in whey proteins (by-products of the dairy industry), which represents 55% of the total whey protein content (15). However, few studies examined the effect of Trp-enriched peptides prepared from *β*-lactoglobulin on diminishing anxiety-related states.

*β*-lactoglobulin is a globular protein with a molecular weight of 18.3 kDa and 162 amino acid residues (16). In addition, it has two disulfide bonds (Cys66–Cys160 and Cys106–Cys199) and a free sulfhydryl group (Cys121) (17). When it is heated or enzymatically hydrolyzed (especially at pH 3.7–5.1), intermolecular disulfide bonds can maintain macromolecular structure and even cause aggregation of proteins, resulting in low enzymatic hydrolysis efficiency of proteins and difficult release of active peptides (18). When the pH was 7.0 and the temperature was 50°C, the *β*-lactoglobulin dimer dissociated into a monomer, and the structure of *β*-lactoglobulin became loose (18, 19). Trp was located between the 19th and 61st amino acids of *β*-lactoglobulin, and the 19th amino acid (Trp) was near the N-terminal, which was easy to expose in monomers and loose structures (16). However, the preparation of *β*-lactoglobulin Trp peptide and its effect on anti-anxiety has been examined rarely.

Accordingly, this study aimed to obtain *β*-lactoglobulin Trp peptides from whey protein using selective proteolysis, which were then preliminarily investigated for the anti-anxiety effect in a *zebrafish* model. Furthermore, we assessed the ability of *β*-lactoglobulin Trp peptide ingestion to enhance 5-HT synthesis, which is an important index of serotonergic activity.

## 2. Materials and methods

### 2.1. Material and chemicals

Acid proteinase from *Aspergillus niger* (activity: 30,000 U/g) was purchased from Donghenghuadao Biotechnology Co., Ltd. (Guangxi, China). Trypsin from porcine pancreas (USP, 1:2,500 U/mg) was purchased from Guangzhou Qiyun Biotechnology Co., Ltd. (Guangdong, China). Whey protein concentrate (WPC with a protein content of 13.45%) was purchased from Guangdong Beric Food Co., Ltd. (Guangdong, China). Commercial synthetic peptides Val-Ala-Gly-Thr-Trp-Tyr, Val-Ala-Gly-Thr-Trp, and Gly-Thr-Trp with 95% purity were purchased from Nanjing Peptide Industry Biotechnology Co., Ltd. (Nanjing, China). *β*-lactoglobulin with 95% purity was purchased from Sigma Co. (St. Louis, MO, USA). The rest of the reagents and chemicals were purchased from Guangzhou Jingke Glass Instrument Co., Ltd. (Guangzhou, China) and were of the analytical grade or better.

### 2.2. Preparation of whey protein

Whey protein was prepared following the alkali-soluble acid precipitation method as described by Zhu et al. (4). The parameters of alkaline extraction were as follows: pH of 11, a ratio of whey protein concentrate (as described previously) to water of 1:10 at 60 ± 1°C for 1 h, centrifugation at 4°C at 8,000 r/min for 20 min, and separation to obtain the supernatant using a centrifuge (GL-21M, Changsha Xiangzhi Centrifuge Instrument Co., Ltd., Changsha, China). The parameters for acid precipitation were as follows: pH of 4.4, centrifugation of 4°C at 8,000 r/min for 20 min, and separation to obtain the precipitate. The precipitate was washed with deionized water at pH 7, vacuum freeze-dried (model: SCIENTZ-18N, Ningbo Scientz Biotechnology Co., Ltd., Ningbo, China), and stored at 4°C for further use. After the alkali-soluble acid precipitation method, the total protein content of the prepared whey protein was 78.25%, and the *β*-lactoglobulin content was 43.46%.

### 2.3. Enriched *β*-lactoglobulin

When *β-*lactoglobulin (two disulfide bonds and a free sulfhydryl group) is heated or enzymatically hydrolyzed under acidic conditions, intermolecular disulfide bonds can maintain macromolecular structure and even cause aggregation of proteins, resulting in low enzymatic hydrolysis efficiency of proteins and difficult release of active peptides (18). However, α-lactalbumin tends to form a “melting ball” state and is easily hydrolyzed by acidic protease under acidic conditions (19). Therefore, this property can be used to selectively hydrolyze α-lactalbumin from whey protein to enriched *β*-lactoglobulin. *β*-lactoglobulin was enriched from whey protein using acid proteinase under the following conditions: pH of 4.0, the ratio of whey protein to water of 1:10, and temperature of 50°C. The reaction mixture was then centrifuged at 8,000 r/min and 4°C for 20 min to obtain the precipitation. The precipitation sample was redissolved in Milli-Q water and analyzed using UPLC. The solution was then loaded onto a ZORBOX Eclipse XDB-C_8_ UPLC column (specifications: 150 × 4.6 mm, 5 μm; Agilent Technologies, USA). The elution was programmed at a flow rate of 0.25 ml/min and room temperature (25°C) for 20 min, with the mobile phase A (10% acetonitrile and 0.1% trifluoroacetic acid) and mobile phase B (90% acetonitrile and 0.1% trifluoroacetic acid), as 0~1 min 35% B; 1 min~5 min 35%~38% B; 5 min~10 min 38%~42% B; 10 min~ 12 min 42%~46% B; 12 min~15 min 46%~90% B; 15 min~18 min 35%~90% B; and 18 min~20 min 35% B. The elution in gradient mode was monitored at 280 nm with a UV detector (UV201, USA). The sample corresponding to the detected peaks of *β*-lactoglobulin was collected and freeze-dried for further investigations.

### 2.4. The preparation of *β*-lactoglobulin Trp peptides

When the pH was 7.0 and the temperature increased to 50°C, the *β*-lactoglobulin dimer dissociated into a monomer, and the structure of *β*-lactoglobulin became loose (18, 19). Trp was located at the 19th (near the N-terminal) and 61st amino acids of *β*-lactoglobulin (which is composed of 162 amino acid residue), and the 19th amino acid (Trp) was easy to be exposed to (16). To obtain Trp peptides (near the N-terminal) of the above-prepared *β*-lactoglobulin, the *β*-lactoglobulin was hydrolyzed using trypsin under the following optimal conditions: pH of 7.0, a ratio of whey protein to water of 1:10, temperature of 50°C, and incubation time of 4 h. The reaction mixture was then centrifuged at 8,000 rpm and 4°C for 20 min using a centrifuge to obtain the supernatants. It was then vacuum-freeze dried and stored at 4°C. The characterization of the Trp peptide prepared from *β*-lactoglobulin was conducted using a UPLC-Q-TOF MS/MS (20). An Agilent 1290 series UPLC system (Agilent Technologies CO., Ltd., Guangzhou, China) was equipped with an Agilent ZORBAX RRHD SB-C_18_ column (2.1 × 50 mm i.d., 1.8 μm, maintained at 30°C). Peptides were analyzed using maXis ImpactTM quadrupole TOF tandem mass spectrometry system (Bruker Daltonics, Beijing, China) fitted with ESI^+^ (Bruker maxis impact) and coupled *via* MS/MS. All the mass spectrometry data were interpreted by peaks automatic sequencing software and *de novo* sequencing software (Bruker Compass Data Analysis 4.1 software) for the identification and quantification of peptides (21). The ion peaks of the main individual peptides were extracted, and the relative contents of the main individual peptides were determined by the ion peak area (20, 22).

### 2.5. Animal trials

The adult *zebrafish* (wild type AB strain, the ratio of ♀ ♂ was 1:1) were bred at the age of 2 months by the *Zebrafish* Laboratory of South China University of Technology. The temperature was 28.5 ± 0.2°C, pH was 7.5 ± 0.02, tank volume was 3 L, the light-dark cycle was 14/10 h, and conductivity was 450~550 μs/cm. The system water is circulating (automatically updated by 10% every day) and is sterilized by an ultraviolet lamp in real-time. *Zebrafish* were bred by an independent breeding system and fed two times a day with abundant shrimp eggs. All experiments involving *zebrafish* were reviewed by the Animal Research Advisory Committee of the South China University of Technology. After a 3-day acclimation period, *zebrafish* were randomly divided into nine groups (*n* = 30 per group) as follows: NC group (the normal control group for treatment with normal water); LAWPH and LAWPL (the group for treatment with *β*-lactoglobulin Trp peptides at a high dose: 500 μg/ml and a low dose: 56 μg/ml); VAGTWYH and VAGTWYL (the group for treatment with Val-Ala-Gly-Thr-Tyr-Trp at a high dose: 500 μg/ml and a low dose: 56 μg/ml); VAGTWH and VAGTWL (the group for treatment with Val-Ala-Gly-Thr-Tyr-Trp at a high dose: 500 μg/ml and a low dose: 56 μg/ml); and GTWH and GTWL (the group for treatment with Gly-Thr-Trp at a high dose: 500 μg/ml and a low dose: 56 μg/ml). The experimental groups (VAGTWY, VAGTW, and GTW) with the *zebrafish* used the synthetic peptides, and the LAWP experimental group with the *zebrafish* used the hydrolysates. The maximum tolerated concentration (MTC: 500 μg/ml) of peptides was determined. In the low-dose group and high-dose group, 1/9 MTC and MTC were selected, respectively.

### 2.6. Behavioral tests

After the fortnight of water-soluble administration, all experimental *zebrafish* were tested for anxiety-like behavior during the daytime (7:00 am−17:00 pm). To obtain accurate results, the whole behavioral testing process was carried out in a quiet and stable environment.

#### 2.6.1. Novel fish tank test

The novel fish tank test was carried out after the fortnight of water-soluble administration. The novel fish tank (a trapezoidal box: bottom length × width × height × top length was 22.5 cm × 7.1 cm × 15.2 cm × 27.9 cm) was filled with water (2 ~ 3 cm above the top surface). Before the experiment, the *zebrafish* were transferred to the novel fish tank and given 10 min to adapt to the surrounding environment. After acclimatization, a 6 min test was started in which the movements/behaviors of *zebrafish* were tracked and recorded using a *zebrafish* behavior trajectory analyzer (*Zebralab* 3.3, Viewpoint, Lyon, France), with the number of times of shuttle up/down and the percentage of time spent in the top half of the tank being measured. At the end of the experiment, the *zebrafish* were removed from the water and returned to the aquarium.

#### 2.6.2. Light-dark test

The model of the light-dark test (length × width × height was 18 × 9 × 7 cm) was divided into two equal parts by a controllable partition board, with one part surrounded by black and the other part by white. The normal water (temperature 28.5 ± 0.2°C, pH 7.5 ± 0.02) was put into the test device for the light-dark preference experiment, the water depth was about 3–4 cm, and *zebrafish* could swim freely during the whole test process. Before the experiment, the *zebrafish* were transferred to the light-dark model for behavioral testing and given 10 min to adapt to the surrounding environment. At the beginning of the test, the *zebrafish* were put gently and quickly into the white area and then a 6 min test was started. The movements/behaviors of *zebrafish* were tracked and recorded using a *zebrafish* behavior trajectory analyzer (*Zebralab* 3.3, Viewpoint, Lyon, France), and the percentage of time spent in the light area was measured and calculated using the following formula:


$$\begin{array}{l} \text{The percentage of time spent in the light area }(\%)\\ =\frac{\text{The time spent in the light area}}{\text{Total test time}}\times 100\% \end{array}$$

### 2.7. Biochemical analyses

After the behavioral tests, the collected brain tissues were frozen in liquid nitrogen for further studies. The concentrations of Trp, 5-HT level, and kynurenine (KYN) were determined by Elisa assay kits following the instructions of the manufacturer (Beijing Solarbio Science and Technology Co., Ltd., Beijing, China). Briefly, 100 mg of brain tissue was homogenized with 9 volumes of phosphate-buffered saline (PBS, pH 7.2) at 6°C using a homogenizer (Xianou-24, Nanjing Xianou Instrument Co., Ltd., Nanjing, China) and centrifuged for 20 min (3,000 r/min, 4°C) to collect the supernatants. The concentrations of Trp, 5-HT level, and KYN of the obtained supernatants from brain tissues were determined using the abovementioned ELISA method. The activity of TPH was determined by Elisa and colorimetric assay kits (Beijing Solarbio Science and Technology Co., Ltd., Beijing, China) as described by Zhu et al. (10) with some modifications.

### 2.8. Statistical analysis

All experimental results were expressed as “mean ± standard deviation” for reporting. The statistical software, Origin 6.1 (Origin Lab Corporation, USA), was used by the primary contributor to the present study. Analysis of variance and significant difference tests were performed using SPSS 16.0 statistical software (SPSS Inc., Chicago, IL, USA). ANOVA was performed to evaluate the difference, followed by the Duncan *post-hoc* test. Statistical significance was defined as a *p*-value of < 0.05.

## 3. Results and discussion

### 3.1. The enrichment of *β*-lactoglobulin

**Figure 1:**
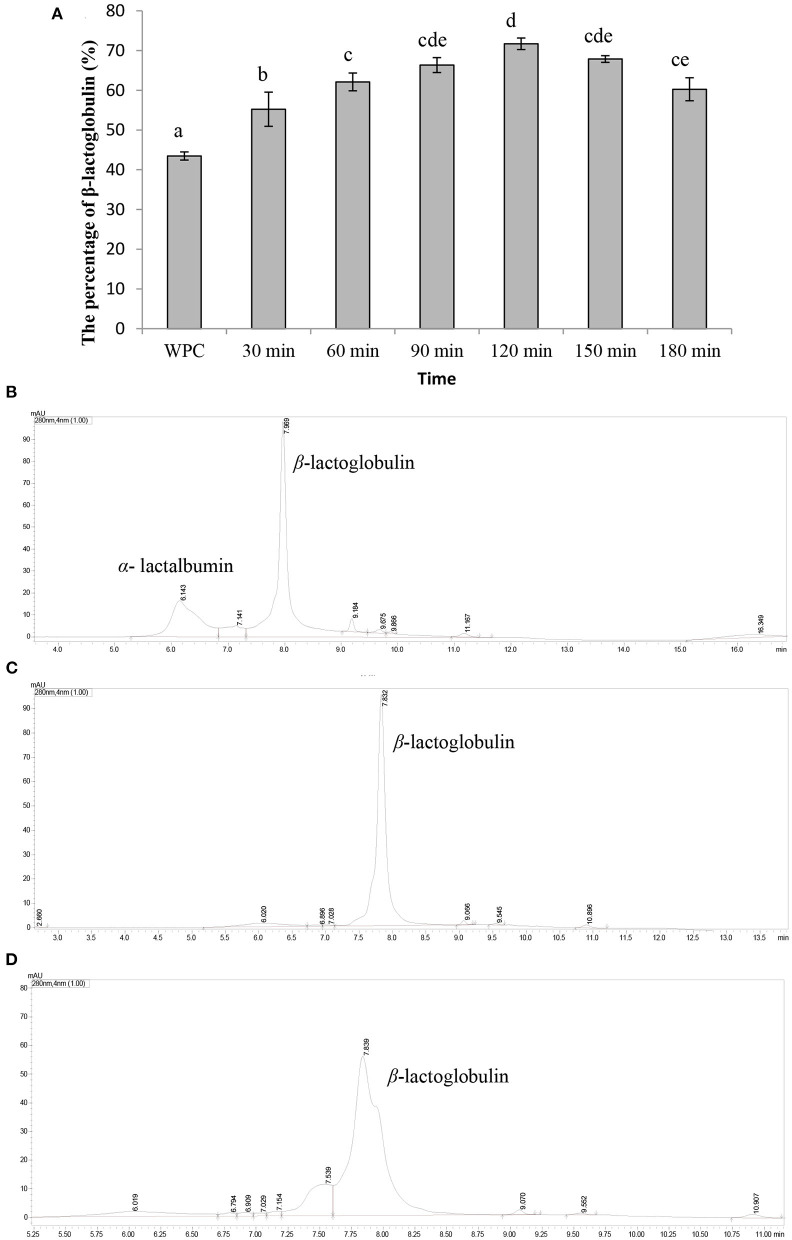
Characterization of *β*-lactoglobulin enriched. Data are expressed as the mean ± SD (*n* = 3), and ANOVA was performed to evaluate the difference, followed by the Duncan *post-hoc* test. **(A)** The percentage of *β*-lactoglobulin. Different letters indicate a significant difference (*p* < 0.05). **(B)** The UPLC spectra of whey protein. **(C)** The UPLC spectrum of whey protein hydrolyzed by acid protease for 120 min. **(D)** The UPLC spectrum of whey protein hydrolyzed by acid protease for 180 min.

The content of *β*-lactoglobulin in whey protein (prepared above) was 43.46 ± 1.02%, as shown in Figures 1A, B. In addition, the content of *β*-lactoglobulin was increased with the hydrolysis time and the content was the highest (71.17 ± 1.45%) when the hydrolysis time was 120 min (Figure 1A). However, as the hydrolysis time continued increasing, the content of *β*-lactoglobulin decreased. As shown in Figure 1C, most of α-lactalbumin in whey protein was hydrolyzed and *β*-lactoglobulin was hardly hydrolyzed when the hydrolysis time was 120 min. Then, *β*-lactoglobulin began to hydrolyze and the degree of hydrolysis gradually increased with the increase in time (Figures 1A, D). Under acidic conditions, α-lactalbumin tends to form a “melting ball” state and was easily hydrolyzed by acidic protease (19). This property can be used to selectively hydrolyze α-lactalbumin from whey protein to enriched *β*-lactoglobulin. Our results showed that α-lactalbumin in whey protein was preferentially hydrolyzed by acidic protease at pH 4.0, which was consistent with previous studies. In summary, the optimal hydrolysis parameters of enriched *β*-lactoglobulin from whey protein by selective hydrolyzed technology were as follows: whey protein concentration of 10%, pH of 4.0, and hydrolysis temperature of 50°C for 120 min.

### 3.2. Characterization of Trp peptides obtained from *β*-lactoglobulin

**Figure 2:**
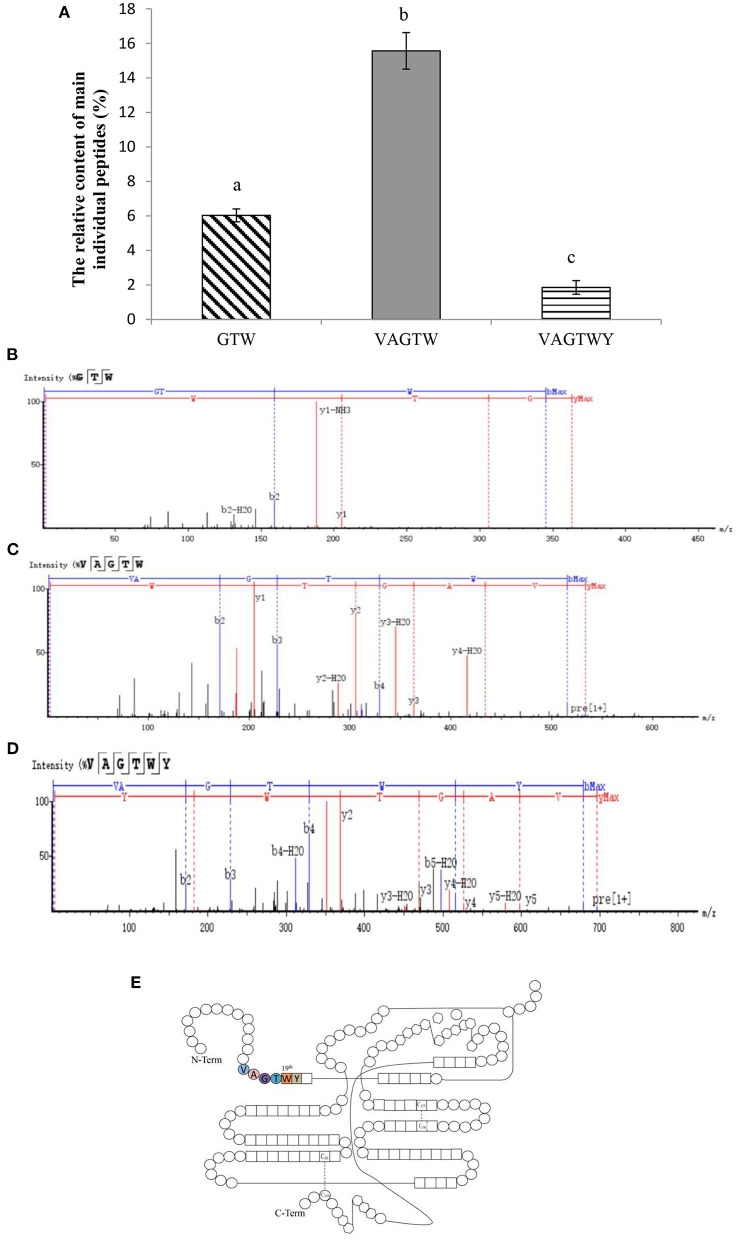
Characterization of Trp peptides obtained from *β*-lactoglobulin. Data are expressed as the mean ± SD, and ANOVA was performed to evaluate the difference, followed by the Duncan *post-hoc test*. **(A)** The relative content of peptides (GTW, VAGTW, and VAGTWT) produced from *β*-lactoglobulin. Different letters (a, b, and c) indicate a significant difference (*p* < 0.05). The UPLC-Q-TOF MS/MS tandem mass spectra (ESI+) of **(B)** GTW (Gly-Thr-Trp), **(C)** VAGTW (Val-Ala-Gly-Thr-Trp), and **(D)** VAGTWY (Val-Ala-Gly-Thr-Trp-Tyr) as interpreted by peaks automatic sequencing software. **(E)** Schematic diagram of the molecular structure of *β*-lactoglobulin.

After 4 h of hydrolysis, trypsin produced a large number of peptides from the *β*-lactoglobulin (enriched above) hydrolysate. Then, three major and novel Trp peptides, Gly-Thr-Trp, Val-Ala-Gly-Thr-Trp, and Val-Ala-Gly-Thr-Trp-Tyr, were identified from the *β*-lactoglobulin hydrolysate (Figure 2A). Trypsin only cleaves K and R (not A, W, and Y) based on the cleavage patterns predicted by ExPASy PeptideCutter (23). Theoretically, GTW and VAGTWY cannot be released from QTMKGLDIQKVAGTWYSLAM; however, these two peptides were identified in our present study, which may be due to miscleavage resulting from weak peptide–protease interaction (23, 24). The total yield of Trp peptides was 23.46% (w/w), and the relative contents of Gly-Thr-Trp, Val-Ala-Gly-Thr-Trp, and Val-Ala-Gly-Thr-Trp-Tyr were 6.03, 15.57, and 1.86% (w/w), respectively. The strong signals detected in positive-ion mode at 363.1659, 533.2725, and 696.3362 m/z corresponded to the ionized peptides Gly-Thr-Trp (Figure 2B), Val-Ala-Gly-Thr-Trp (Figure 2C), and Val-Ala-Gly-Thr-Trp-Tyr (Figure 2D). Counting from the N-Term, the sequences of Trp peptides prepared from *β*-lactoglobulin were all located at the position between the 15th and 20th amino acids (Figure 2E). In addition, the three peptides identified were all made up of Trp peptides (the 19th amino acid of *β*-lactoglobulin). Based on these obtained results, trypsin could hydrolyze *β*-lactoglobulin to produce Trp peptides consisting of 19th tryptophan.

### 3.3. Effects of Trp peptides on behaviors of zebrafish in the novel fish tank test

Anxiety-like behaviors in the novel fish tank test after the fortnight of treatment with Trp peptides are summarized in Table 1. The result shows that anxiety-like behaviors were all decreased for the Trp peptide treatment groups compared with the NC *zebrafish* (Table 1). Notably, the number of times of shuttle up/down (14.24 ± 4.78) in the NC group was significantly (*p* < 0.05) lower than that in all the Trp peptide treatment groups. The number of times of shuttle up/down in the VAGTWH group (26.17 ± 4.66) was significantly higher (*p* < 0.05) compared to the LAWP (H and L), GTW (H and L), and VAGTWY (H and L) groups. The number of times of shuttle up/down was decreased in the following order: VAGTWH > VAGTWL > GTWL > GTWH > LAWPH > VAGTWYH > LAWPL > VAGTWYL > NC. The percentage of retention time in the top half of the tank increased significantly (*p* < 0.05) in the LAWPH, GTW (H and L), and VAGTW (H and L) groups compared to the NC group (22.52 ± 1.43%). However, there was no significant difference (*p* > 0.05) in the LAWPL and VAGTWY (H and L) groups in the percentage of retention time in the top half of the tank compared to the NC group. Similarly, the percentage of retention time in the top half of the tank in the VAGTWH group was also significantly higher (*p* < 0.05) than all the other groups. Hawkey et al. (25) showed that the novel fish tank test was a direct, repeatable method for measuring *zebrafish* anxiety-like behaviors. When placed in a novel tank, *zebrafish* often dive to the bottom of the tank (avoidance). *Zebrafish* will also be stimulated by a novel area (top of a fish tank) and develop an urge to explore; thus, *zebrafish* form an anxious state of avoidance/exploration. According to previous studies, our results indicated that *β*-lactoglobulin Trp peptides could alleviate anxiety-like behaviors of *zebrafish* in the novel fish tank test after the fortnight treatment (effectiveness: VAGTW > GTW > VAGTWY).

**Table 1:** Effect of Trp peptide on behaviors of *zebrafish* in novel fish tank test (*n* = 30).

**Group**	**The number of times of shuttle up/down**	**Percentage of retention time in the top half of the tank (%)**
NC	14.24 ± 4.78^a^	22.52 ± 1.43^a^
LAWPH	20.28 ± 3.45^bc^	29.17 ± 3.03^bd^
LAWPL	19.48 ± 2.16^bc^	26.32 ± 2.81^ab^
GTWH	21.37 ± 2.76^cd^	36.30 ± 1.90^c^
GTWL	22.40 ± 3.14^cd^	34.07 ± 2.28^cd^
VAGTWH	26.17 ± 4.66^e^	42.28 ± 3.86^e^
VAGTWLV	23.89 ± 3.15^de^	39.15 ± 4.83^ce^
VAGTWYH	20.13 ± 2.52^bc^	27.52 ± 3.17^ab^
VAGTWYL	17.75 ± 3.71^b^	26.55 ± 2.16^ab^

All data were expressed as mean ± SD. ANOVA was performed to evaluate the difference, followed by the Duncan post-hoc test. A p-value of < 0.05 denotes the statistical significance of the data. Different letters within the same column indicate significant differences.

### 3.4. Effects of Trp peptides on behaviors of zebrafish in the light-dark test

The light-dark test, a kind of anxiety stress model, is a classical method for screening anti-anxiety drugs and establishing and evaluating the *zebrafish* anxiety model, which has been widely used in basic research in stress response, psychopharmacology, or neuropharmacology (26, 27). Anxiety-like behaviors in the light/dark test after the fortnight treatment with Trp peptides are summarized in Table 2. After the fortnight treatment of this study, the number of times of shuttling light/dark (30.33 ± 2.08) in the NC *zebrafish* was significantly lower (*p* < 0.05) than that in the Trp peptide treatment groups (Table 2). However, the number of occasions of shuttling light/dark in the VAGTWH (43.08 ± 3.97) group was significantly higher (*p* < 0.05) than all the other groups. Notably, the difference in the number of times of shuttling light/dark among the high or low dose of the LAWP, GTW, and VAGTWY groups was insignificant (*p* > 0.05); however, Trp peptide supplementation increased the number of times of shuttle light/dark all in a dose-dependent manner. Except for the LAWPL and VAGTWYL groups, the percentage of time spent in the light area was significantly (*p* < 0.05) lower in the NC group compared to all the other groups (22.52 ± 1.43%). The percentage of time spent in the light area was slightly higher in the VAGTWH group, but there was no significant difference (*p* > 0.05) among the LAWPH, GTW (H and L), VAGTW (H and L), and VAGTWYH groups. Trp peptide supplementation increased the percentage of time spent in the light area, all in a dose-dependent manner. Studies that employed the light-dark task found that animals exhibit a strong preference for the dark compared to the light compartment, and higher levels of anxiety-like behaviors are associated with more time spent in the dark compartment (26, 27). Accordingly, our results indicated that *β*-lactoglobulin Trp peptide ingestion could reduce the anxiety-like behaviors of *zebrafish* in the light-dark test in a dose-dependent manner. In terms of anti-anxiety behaviors, VAGTW seemed more effective (*p* < 0.05) than GTW and VAGTWY.

**Table 2:** Effect of Trp peptide on behaviors of *zebrafish* in the light-dark test (*n* = 30).

**Group**	**The number of times of shuttle light/dark**	**Percentage of time spent in the light area (%)**
NC	30.33 ± 2.08^a^	26.04 ± 4.22^a^
LAWPH	38.85 ± 1.87^bc^	38.05 ± 3.29^bc^
LAWPL	37.25 ± 3.44^bc^	33.36 ± 3.83^ab^
GTWH	38.94 ± 3.71^bc^	36.65 ± 5.81^bc^
GTWL	38.26 ± 2.88^bc^	35.56 ± 4.66^bc^
VAGTWH	43.08 ± 3.97^d^	42.66 ± 3.42^c^
VAGTWLV	40.02 ± 3.49^cd^	39.54 ± 3.63^bc^
VAGTWYH	36.41 ± 2.11^bc^	34.81 ± 2.71^bc^
VAGTWYL	35.82 ± 2.83^b^	31.35 ± 5.60^ab^

All data were expressed as mean ± SD. ANOVA was performed to evaluate the difference, followed by the Duncan post-hoc test. A p-value of < 0.05 denotes the statistical significance of the data. Different letters within the same column indicate significant differences.

### 3.5. Effect of Trp peptides on the activity of THP and level of 5-HT in zebrafish

**Figure 3:**
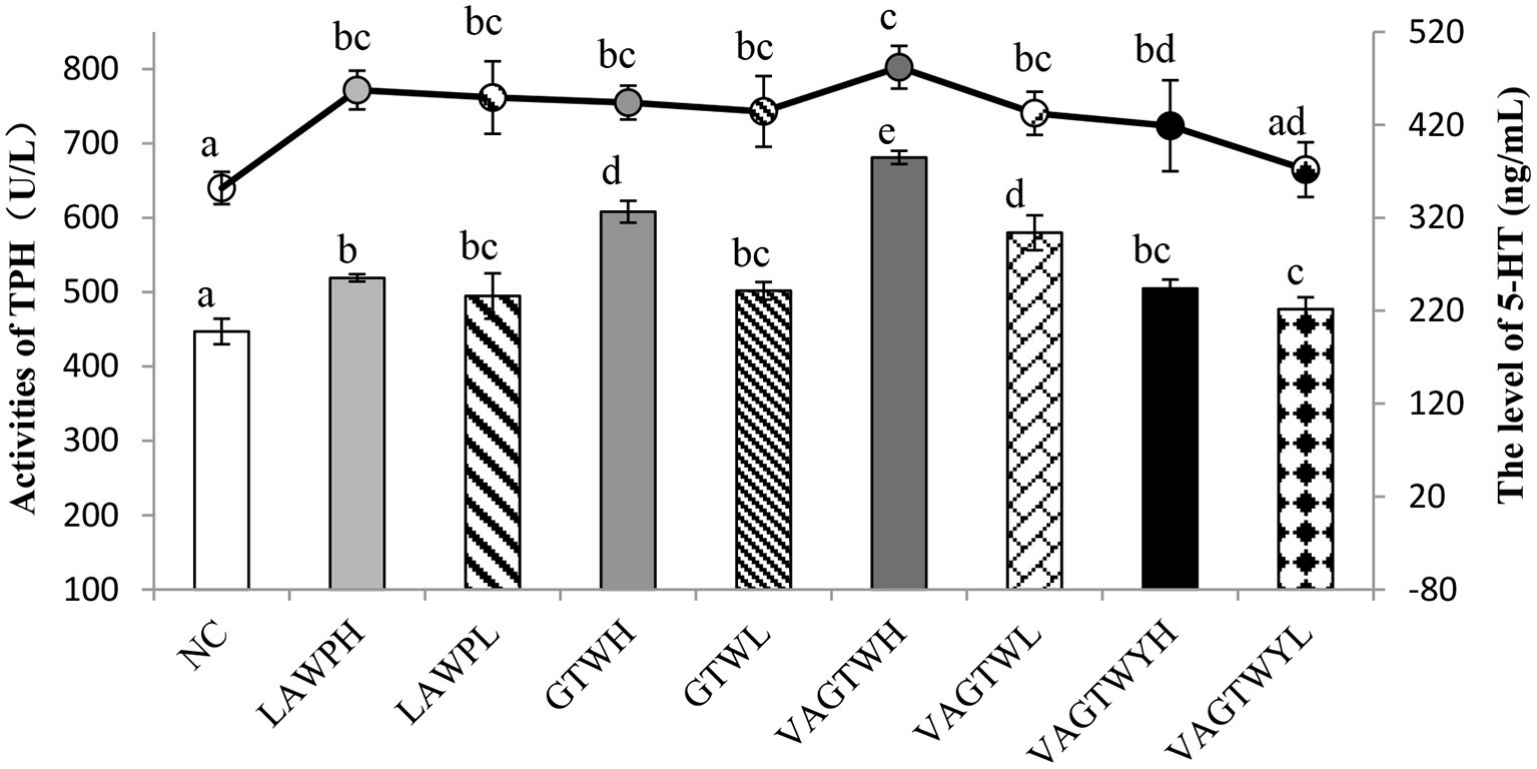
Effect of Trp peptides on the activity of THP and level of 5-HT in *zebrafish*. NC group (the normal control group for treatment with normal water); LAWPH (the group for treatment with *β*-lactoglobulin Trp peptides at a high dose: 500 μg/ml); LAWPL (the group for treatment with *β*-lactoglobulin Trp peptides at a low dose: 56 μg/ml); VAGTWYH (the group for treatment with Val-Ala-Gly-Thr-Tyr-Trp at a high dose: 500 μg/ml); VAGTWYL (the group for treatment with Val-Ala-Gly-Thr-Tyr-Trp at a low dose: 56 μg/ml); VAGTWH (the group for treatment with Val-Ala-Gly-Thr-Tyr-Trp at a high dose: 500 μg/ml); VAGTWL (the group for treatment with Val-Ala-Gly-Thr-Tyr-Trp at a low dose: 56 μg/ml); GTWH (the group for treatment with Gly-Thr-Trp at a high dose: 500 μg/ml); GTWL (the group for treatment with Gly-Thr-Trp at a low dose: 56 μg/ml). Data are expressed as the mean ± SD (*n* = 30), and ANOVA was performed to evaluate the difference, followed by the Duncan *post-hoc* test. The bar graph and the line graph show the activities of TPH (the left ordinate) and the level of 5-HT (the right ordinate), respectively. Different letters indicate a significant difference (*p* < 0.05).

After the administration process, the activity of THP was significantly lower (*p* < 0.05) in the normal control (447.07 U/L) group than in all Trp peptide treatment groups (Figure 3). The activity of TPH in the VAGTWH group was significantly (*p* < 0.05) higher than all the other groups. The activity of TPH in the GTWH and VAGTWL groups was significantly (*p* < 0.05) higher than the LAWP (H and L), GTWL, and VAGTWY (H and L) groups, while they were significantly (*p* < 0.05) lower than the VAGTWH group. Nevertheless, there was no significant difference (*p* > 0.05) among the LAWP (H and L), GTWL, and VAGTWY (H and L) groups. The level of 5-HT was significantly lower (*p* < 0.05) in the NC (352.10 ng/ml) group than in all Trp peptide treatment groups, except in the VAGTWYH group. The level of 5-HT was slightly higher in the VAGTWH group, but there was no significant difference (*p* > 0.05) among the LAWP (H and L), GTW (H and L), and VAGTW (H and L) groups. Notably, the level of 5-HT in the VAGTWH group was significantly higher (*p* > 0.05) than that in the VAGTWY (H and L) group. As the key rate-limiting enzyme, the activity of TPH in brain tissue directly affects the level of 5-HT in the central nervous system (28). The abnormal level of 5-HT in the brain is closely related to anxiety disorders (28, 29). Previous studies showed that most anti-anxiety drugs act by influencing the neurotransmitter, including the increase in the levels of 5-HT in the brain (10). According to the results of this study, Trp peptides had stimulatory effects on the level of 5-HT, and such effects likely occurred *via* boosting the activation of TPH.

### 3.6. Effect of Trp peptides on Trp catabolite

After the fortnightly treatment of this study, the NC group had a normal Trp concentration, and 5-HT/Trp (1,562.88) and KYN/Trp (1,421.52) ratios in brain tissues for the group have been indicated in Table 3. The concentrations of Trp in the LAWP (H and L), GTWH, VAGTW (H and L), and VAGTWYH groups were significantly higher (*p* < 0.05) than those in the NC, GTWL, and VAGTWYL groups. The concentrations of Trp in the GTWL and VAGTWYL groups were slightly higher, but there was no significant difference (*p* > 0.05) compared with the NC group. Excepting for the VAGTWYL group, the ratio of 5-HT/Trp in the NC group was significantly lower (*p* < 0.05) than that in all other Trp peptide treatment groups. The ratio of 5-HT/Trp decreased in the following order: GTWL > LAWPL > VAGTWH > LAWPH > GTWH > VAGTWL > VAGTWYH > VAGTWYL > NC. However, the ratio of KYN/Trp in the NC group was significantly higher (*p* < 0.05) than in all Trp peptide treatment groups, except the GTWL and VAGTWYL groups. Additionally, the ratio of KYN/Trp increased in the following order: LAWPH < VAGTWYH < GTWH < VAGTWH < VAGTWL < LAWPL < GTWL < VAGTWYL < NC. These results suggest that Trp peptides significantly increased the level of 5-HT in the *zebrafish*, possibly by influencing Trp concentration, 5-HT/Trp ratio, and KYN/Trp ratio of brain tissue. A large number of studies showed that Trp and its metabolite were critical for monitoring anxiety disorders (30, 31), and a positive relationship was found between dietary Trp and the level of 5-HT in the brain (7). Our results were consistent with those of previous studies that showed that ingestion of Trp peptide diets did, in fact, increased the concentration of Trp in brain tissues, with VAGTWH being more effective. Previous research showed that the ratio of 5-HT/Trp was reduced and the ratio of KYN/Trp was increased in nervous and mental diseases (such as anxiety or depression) (32). Our result was consistent with the previous studies that showed that ingestion of Trp peptide diets, in fact, enhanced the ratio of 5-HT/Trp, with VAGTWH being more effective. Thus, Trp peptide might act more effectively in anti-anxiety *via* enhancing the concentration of Trp and the ratio of 5-HT/Trp and reducing the ratio of KYN/Trp.

**Table 3:** The effect of Trp peptides on Trp catabolite.

**Group**	**The concentration of Trp (pg/mL)**	**The ratio of 5-HT/Trp**	**The ratio of KYN/Trp**
NC	225.29 ± 15.78^a^	1562.88 ± 132.15^a^	1421.52 ± 86.45^a^
LAWPH	267.33 ± 23.34^bc^	1711.53 ± 229.36^bc^	1191.27 ± 105.83^b^
LAWPL	257.43 ± 24.72^b^	1745.89 ± 183.42^b^	1263.00 ± 132.15^b^
GTWH	264.86 ± 16.53^bc^	1676.62 ± 158.33^c^	1236.41 ± 111.43^b^
GTWL	248.01 ± 13.27^ab^	1751.88 ± 246.16^b^	1379.24 ± 93.62^a^
VAGTWH	278.78 ± 57.86^c^	1729.16 ± 134.52^b^	1248.71 ± 145.41^b^
VAGTWL	263.69 ± 15.02^bc^	1640.15 ± 133.24^cd^	1254.42 ± 73.08^b^
VAGTWYH	259.30 ± 16.84^b^	1616.14 ± 127.40^d^	1212.13 ± 126.42^b^
VAGTWYL	236.27 ± 15.01^ab^	1573.94 ± 156.16^ad^	1419.81 ± 81.26^a^

All data were expressed as mean ± SD. ANOVA was performed to evaluate the difference, followed by the Duncan post-hoc test. A p-value of < 0.05 denotes the statistical significance of the data. Different letters within the same column indicate significant differences.

## 4. Conclusion

This study has demonstrated the feasibility of enriching *β*-lactoglobulin in whey protein by selective hydrolysis technique. Then, three Trp peptides (Val-Ala-Gly-Thr-Trp-Tyr, Val-Ala-Gly-Thr-Trp, and Gly-Thr-Trp) were first produced from *β*-lactoglobulin and identified using UPLC-Q-TOF-MS/MS. This study also supports the effect of *β*-lactoglobulin Trp peptide on anti-anxiety. *β*-lactoglobulin Trp peptide, Val-Ala-Gly-Thr-Trp-Tyr, Val-Ala-Gly-Thr-Trp, and Gly-Thr-Trp at two respective doses were examined and found to decrease anxiety-like behaviors, increase Trp concentration (the precursor of 5-HT), and increase the activity of THP (the key rate-limiting enzyme of the serotonin pathway) while enhancing serotonin (5-HT) synthesis and the ratio of 5-HT/Trp, to different extents, in the brain tissue. The Val-Ala-Gly-Thr-Trp might act more effectively on anxiety *via* enhancing the level of 5-HT and the ratio of 5-HT/Trp as compared to the other two Trp peptides. In conclusion, our result showed that ingesting *β*-lactoglobulin Trp peptide affects anxiety behaviors and 5-HT levels of *zebrafish*. The potential anti-anxiety functions of *β*-lactoglobulin Trp peptide may be associated with increasing TPH activity and enhancing 5-HT synthesis, with the Val-Ala-Gly-Thr-Trp being more effective.

## Data availability statement

The original contributions presented in the study are included in the article/supplementary material, further inquiries can be directed to the corresponding authors.

## Ethics statement

The animal study was reviewed and approved by the Animal Research Advisory Committee of South China University of Technology and followed the National Institutes of Health Guidelines.

## Author contributions

XZ, DX, and CC completed conceptualization and formal analysis. XZ and CC performed the experiments and analyzed the data. CC acquired funding. XZ completed the writing-original draft. XZ, DX, QZ, and YL completed writing-review and editing, with input from all authors. All authors contributed to the article and approved the submitted version.
